# Investigating the Targeting Power to Brain Tissues of Intranasal Rasagiline Mesylate-Loaded Transferosomal In Situ Gel for Efficient Treatment of Parkinson’s Disease

**DOI:** 10.3390/pharmaceutics15020533

**Published:** 2023-02-05

**Authors:** Hala N. ElShagea, Rana R. Makar, Alaa H. Salama, Nermeen A. Elkasabgy, Emad B. Basalious

**Affiliations:** 1 Department of Pharmaceutics and Industrial Pharmacy, Faculty of Pharmacy, Ahram Canadian University, 6th of October City, Cairo 12451, Egypt; 2 Pharmaceutical Technology Department, National Research Centre, Dokki, Cairo 12622, Egypt; 3 Department of Pharmaceutics and Industrial Pharmacy, Faculty of Pharmacy, Cairo University, Kasr El-Aini Street, Cairo 11562, Egypt

**Keywords:** drug delivery, nanotechnology, mucoadhesion, nose-to-brain delivery, brain kinetics

## Abstract

Rasagiline mesylate (RSM) is a hydrophilic drug with poor oral bioavailability (36%) because of hepatic first-pass metabolism. The present study focuses on delivering RSM directly to the brain through its inclusion within transferosomal in situ gel administered through the intranasal (IN) route. Transferosomes were formed by the thin-film hydration method with the aid of Design-Expert^®^ software by varying the edge activator (EA) type in the absence or presence of cholesterol. By desirability calculations, the optimum formulation was composed of phosphatidylcholine and sodium deoxycholate as an EA (5:1% *w*/*w*) with no cholesterol. The optimum formulation was 198.63 ± 34.98 nm in size and displayed an entrapment efficiency of 95.73 ± 0.09%. Transmission electron microscopy revealed discrete and spherical vesicles. Optimized transferosomes were further incorporated into an in situ gel composed of 0.5% pectin, 15% Pluronic^®^ F-127, and 5% Pluronic^®^ F-68 and tested for the in vivo performance. The systemic as well as brain kinetics were assessed in rats by comparing the IN-administered in situ gel to the IV aqueous solution. The optimum in situ gel showed safety and biocompatibility on rats’ nasal mucosa with enhanced brain bioavailability (131.17%). Drug targeting efficiency and direct transport percentage indices (304.53% and 67.16%, respectively) supported successful brain targeting offering direct nose-to-brain drug delivery.

## 1. Introduction

Parkinson’s disease is a progressive neurological disorder in which the neurons of the substantia nigra pars compacta and the nigrostriatal tract are degenerated [1]. Additionally, it is accompanied by a decrease in dopamine levels in the striatum, which causes motor dysfunction [2]. It targets about 2% of people over the age of 60 years [3]. It is thought that genetic and environmental factors play a significant role in the onset and progression of Parkinson’s disease [4]. Tremors, spasms, muscle rigidity, bradykinesia, hypersensitivity, abnormal posture, and movement are among the specific motor symptoms caused by the dopaminergic neurons loss, which can affect patients’ quality of life [5]. Parkinson’s disease leads to a decline in life expectancy based on the severity of motor impairment. So, in order to reduce the mortality rate, several approaches are used, including effective therapy and deep brain stimulation [6].

Rasagiline mesylate (RSM) is a second-generation monoamine oxidase inhibitor [7,8]. Unlike selegiline (the first monoamine oxidase inhibitor used for Parkinson’s disease treatment), RSM does not produce amphetamine-like metabolites and is not accompanied by the neurotoxic side effects caused by these metabolites [9]. However, RSM produces a neuroprotective metabolite called L-aminoindane, which in turn leads to a lower recommended therapeutic daily dose [10]. RSM was introduced into the market as oral tablets (Azilect^®^) with a single daily recommended dose of 1 mg [11]. RSM possesses reduced oral bioavailability (36%) because of the extensive first-pass metabolism [12], which affects its therapeutic efficacy. Thus, altering the route of administration is one of the promising solutions to boost the drug bioavailability and efficacy.

The intranasal (IN) route of administration is considered a non-invasive path to deliver the drug directly to the central nervous system [13]. The IN route is characterized with the ease of self-administration, which in turn enhances patient compliance [14]. Additionally, it allows the direct access of the drug to the brain, bypassing the hepatic first-pass metabolism that occurs upon the conventional oral administration. Intranasally administered drugs can escape the blood–brain barrier (BBB) that limits drug transportation to the brain, leading to a significant enhancement in drug bioavailability [15,16,17]. Direct nose-to-brain targeting can occur through trigeminal and olfactory pathways that are found in the nasal cavity, thus reaching the brain in sufficient concentrations [18].

However, the limiting factors for IN absorption in general are the reduced capacity of the nasal cavity and rapid mucociliary clearance [19]. Hence, the use of mucoadhesive agents with or without the addition of permeation enhancers can be adopted for extending the mucociliary residence time, which in turn enhances drug absorption and bioavailability. This enhancement in drug bioavailability is mainly linked to the increased residence time in the nasal cavity [20]. In case of delivering hydrophilic drugs, another limiting factor for IN absorption is added, which is the low permeability of hydrophilic molecules [21]. This can be addressed by rendering the drug more lipophilic through the inclusion of lipophilic vesicles, which can boost the IN permeability of the hydrophilic drugs [22]. In conclusion, formulating an IN-administered formulation loaded with a hydrophilic drug with enhanced drug bioavailability is considered a challenge.

Several approaches were adopted in order to reduce the mucociliary clearance and increase the nasal residence time. Among these approaches is the preparation of in situ-forming gels [23], where the drug is administered in solution form and transforms into gel in response to some endogenous stimuli, such as the increase in temperature or pH, or the presence of ions [24,25]. Combining in situ-forming gel polymers with mucoadhesive polymers results in a greater effectiveness and higher efficiency through the prolongation of the residence time at the administration site, which in turn enhances the drug bioavailability [26,27,28,29]. An optimum concentrations selection of the used polymers is based on the sol-to-gel transition temperatures and gelation time [30]. Sherje et al. was able to formulate cyclodextrin-based in situ gel (pH-responsive) of paliperidone using different polymers, such as Carbopol 934 and hydroxypropyl methyl cellulose K4M, which were capable of transforming into gel at 34 °C after 2.1 ± 0.1 min, where the gelation time was sufficient to resist the rapid mucociliary clearance [31]. On the other hand, Liu et al. succeeded in the preparation of Pluronic-based in situ hydrogels for vaginal application of nonoxinol-9 using 18% Pluronic^®^ F-127 and 6% Pluronic^®^ F-68, which transformed into gel in the temperature range of 28.5–31.5 °C [32].

Nanosized drug delivery systems may also be adopted to enhance drug permeability across biological membranes due to the small particle size and large surface-area-to-mass ratio of the formed particles, which in turn improve the therapeutic efficacy [33,34]. This is in addition to their capability of carrying both hydrophilic and lipophilic drugs, making them available for various routes of administration [35]. Transferosomes are liposome-derived nanovesicles that have an ultra-flexible nature owing to the incorporation of edge activators (EA). Transferosomes have the ability to squeeze through the nasal mucosa due to their elastic nature [36]. EA are single-chain surfactants that increase the deformability of the formed vesicles through destabilization of the lipid bilayers, resulting in a higher capability to cross the biological membranes [37]. In addition to their general advantages as a nanocarrier, transferosomes are considered superior drug carriers for IN delivery as they were reported to enhance membrane permeability and decrease mucociliary clearance, as well as reduce nasal peptidase degradation, which in turn led to the enhanced drug bioavailability of IN-administered drugs [38].

The literature includes some studies addressing the direct delivery of the RSM to the brain via the IN route. One study discussed the preparation of RSM-loaded solid lipid nanoparticles by conducting a modified microemulsion method. The selected formulation possessed an entrapment efficiency value of 37.8%. The selected formulation was further incorporated into a thermosensitive mucoadhesive gel prepared using Pluronic^®^ F-127 and hydroxypropyl methylcellulose E5. Ex vivo permeation studies showed the superiority of the solid lipid nanoparticles-loaded in situ gel over the drug suspension and free drug-loaded in situ gel. However, this study lacked the investigation of the in vivo behavior on an animal model [39]. Another study discussed the preparation of RSM-loaded deformable ethosomes. The prepared ethosomes possessed an entrapment efficiency value of 38%. The authors indicated the success of the prepared ethosomes to permeate through the nasal mucosa. Nonetheless, the study lacked the pre-clinical investigations of the proposed formulation [40]. From the previous studies, we observed that the obtained entrapment efficiency values were less than 40%, which is considered a low value and is accredited to the drug’s aqueous nature [41]. On the other hand, the present study succeeded in enhancing the proportion of the entrapped drug in the prepared transferosomes to values exceeding 95%, which was considered challenging when incorporating a hydrophilic drug in lipid-based vesicles. For the authors’ information, this is considered the first study discussing the loading of RSM in transferosomes intended for brain delivery through the intranasal route. Moreover, the current study avoided the use of harmful organic solvents, such as chloroform, and presented the preparation of the transferosomal formulation using ethanol. Moreover, this research is considered a comprehensive study that combines an in vitro characterization of the delivery system and in vivo investigation into an animal model (rats) to determine the efficacy of brain targeting.

This study aimed at exploring the pairing of the used lipid component (phosphatidylcholine) and EA for the formulation of RSM-loaded transferosomes. Following this, the selection of the most suitable combination of thermosensitive and mucoadhesive polymers for the preparation of an in situ gel to be later loaded with the optimum transferosomal formulation was performed. The transferosomal formulation and the loaded in situ gel are characterized for choosing the best formulation. The ability of the formed in situ gel to form gel at physiological temperature, its rheological properties, and in vitro drug release were assessed. Finally, the therapeutic efficacy of the optimum in situ gel transferosomal formulation was evaluated in vivo using Wistar Albino rats for assessing brain kinetics in addition to brain-targeting indices to ensure nose-to-brain drug targeting in sufficient concentrations.

## 2. Materials and Methods

### 2.1. Materials

Rasagiline mesylate (RSM) was kindly gifted by Marcyrl Pharmaceutical Industries (Cairo, Egypt). Phosphatidylcholine from egg yolk was obtained from Fisher Scientific (Hampton, NH, USA). Absolute ethanol, Span^®^ 60, sodium cholate, sodium deoxycholate, Pluronics (F-68, L-31, L-35), and cholesterol were procured from Sigma-Aldrich (St. Louis, MO, USA). Potassium dihydrogen phosphate and disodium hydrogen phosphate were provided by El-Nasr Pharmaceutical Chemicals Company (Cairo, Egypt). Dialysis bag with cutoff 12–14 KDa with flat width of 25 mm was obtained from FREY scientific, Nashua, NH, USA, as well as calcium ammonium nitrate, ammonium formate, and ethyl acetate (LC-MS grade). All other chemicals were of reagent grade and used as received.

### 2.2. Methodology

#### 2.2.1. Preparation of RSM-Loaded Transferosomes

RSM-loaded transferosomes were prepared by thin-film hydration technique to enhance the drug incorporation in the transferosomal vesicles [42]. In a round-bottom flask, 33.33 mg edge activator (EA), 166.66 mg phosphatidylcholine, and cholesterol, if present, were mixed and dissolved together with 10 mg RSM in 15 mL ethanol. Evaporation of the organic solvent was carried out under vacuum at 50 °C using rotary evaporator (Heidolph VV 2000, Burladingen, Germany) at rotation speed 90 rpm until a thin film was deposited on the inner wall of the round-bottom flask. The formed film was then hydrated with 10 mL phosphate-buffered saline (PBS; pH 7.4) under normal pressure and temperature 50 °C. Following, the obtained dispersions were sonicated (Elmasonic S30H, Singen, Germany) for 2 min to ensure that the formed dispersions were free from any aggregates and then stored overnight at 4 °C. Different transferosomal formulations were prepared by altering the type of EA in presence and absence of cholesterol according to a mixed factorial design (6^1^.2^1^) (Table 1). Blank transferosomes were prepared concurrently using the same weights but without the addition of drug to eliminate any interactions from the used ingredients [43].

The construction and analysis of the adopted experimental design were performed using Design-Expert^®^ software (Stat-Ease, Inc., Minneapolis, MN, USA) so as to ascertain the impact of various variables on the characteristics of the formulated RSM-loaded transferosomes. The desirability function was used in order to determine the optimum preparation condition. The independent variables were the type of EA (sodium cholate, sodium deoxycholate, Pluronic^®^ F-68, Pluronic^®^ L-35, Pluronic^®^ L-31, and Span^®^ 60) [X1] with the presence/absence of cholesterol [X2]. On the other hand, the dependent variables were VS [Y1], ZP [Y2], and %EE [Y3]. In order to determine the optimized formulation, Y1 had to be minimized, while Y2 and Y3 had to be maximized. The formulation with the highest desirability was considered as the optimum formulation and selected for further study. The composition of the prepared transferosomal formulations is clarified in Table 2.

#### 2.2.2. Characterization of RSM-Loaded Transferosomes

##### Assessment of Vesicle Size (VS), Polydispersity Index (PDI), and Zeta Potential (ZP)

VS, PDI, and ZP are crucial parameters for the selection of the optimized transferosomal formulation, and they have an impact on the stability and quality of the formulated transferosomes. These parameters were measured using ZetaSizer Nano ZS (Malvern Instrument Ltd., Worcestershire, UK). Each of the prepared formulations was diluted with distilled water (1:10 *v*/*v*) until obtaining a faint opalescent appearance with suitable intensity for light scattering. The measurements were performed in duplicates, and the outcomes were expressed as mean ± SD.

##### Assessment of Entrapment Efficiency (%EE)

The incorporation of water-soluble drugs in transferosomes (lipid-rich vesicles) is one of the main challenges in the formulation of such vesicles. Assessment of %EE was carried out using the indirect method such that 1.5 mL of the prepared RSM-loaded transferosomal dispersions were ultra-centrifuged at 18,000 rpm at 4 °C for 1 h using a cooling centrifuge (Beckman, Fullerton, Canada). The supernatant was filtered through Millipore^®^ filter (0.45 µm) and assessed spectrophotometrically at 271 nm for the determination of the amount of free RSM (unentrapped RSM) (Schimadzu spectrophotometer, model UV-1601 PC, Kyoto, Japan). The same procedures were carried out for the blank transferosomal dispersions to exclude any possible interferences [43]. The test was executed in duplicates, and the readings were stated as mean ± SD. The subsequent equation was utilized for calculating the %EE [43]:

(1) $$\%EE=\frac{\mathrm{Total}\;\mathrm{amount}\;\mathrm{of}\;\mathrm{RSM}\;–\;\mathrm{Free}\;\mathrm{RSM}}{\mathrm{Total}\;\mathrm{amount}\;\mathrm{of}\;\mathrm{RSM}}\times 100$$

#### 2.2.3. Characterization of the Optimized Transferosomal Formulation

##### Fourier Transform Infrared Spectroscopy (FTIR)

FTIR spectrophotometer (IRspirit, Shimadzu, Kyoto, Japan) was used for the determination of any possible interactions between the different components of the optimized RSM-loaded transferosomal formulation. This process was carried out at ambient temperature by placing about 2–3 mg of each individual component, in addition to the freeze-dried formulation (F12), followed by scanning at the range of 4400–400 cm^−1^. The samples were scanned with scanning number of 32 scans per sample at a resolution of 4 cm^−1^. Freeze-drying process was carried out such that samples were placed in glass containers and frozen at −80 °C then transferred to the lyophilizer (Novalyphe-NL 500; Savant Instruments Corp, Holbrook, NY, USA). The process continued for 48 h at freezing temperature of −50 °C under vacuum pressure 0.001 mbar.

##### Transmission Electron Microscopy (TEM)

TEM was conducted for the detailed morphological examination of the optimized RSM-loaded transferosomal formulation by negative staining technique. One drop of the aqueous dispersion was added on carbon-coated copper grid, left to adhere for 1 min, then excess dispersion was removed with a filter paper. Following this, 1% phosphotungestic acid solution was added and left to dry with the sample at room temperature before being examined at 100 kV [44,45].

#### 2.2.4. Preparation of RSM-Loaded Transferosomal In Situ Gels

The cold technique described by Zaki et al. [46] was followed for the preparation of in situ gel containing the optimized RSM-loaded transferosomal formulation. Different concentrations of the thermosensitive polymers, such as Pluronic^®^ F-127 and Pluronic^®^ F-68, in addition to the mucoadhesive pectin were used for the formulation of the in situ gel. Using a magnetic stirrer (Jenway, Staffordshire, England), 0.5% *w*/*v* of pectin was dispersed in 5 mL of the optimized transferosomal formulation, followed by the addition of a mixture of Pluronic^®^ F-127 and Pluronic^®^ F-68 in different concentrations with continuous stirring at 300 rpm [39]. The prepared dispersions were kept in the refrigerator overnight until clear solutions were obtained [26]. The composition of the formulated in situ gels is compiled in Table 3.

#### 2.2.5. Characterization of the Prepared RSM-Loaded Transferosomal In Situ Gels

##### Determination of Sol-to-Gel Transition Temperature and Gelation Time

The in situ gels were tested for their sol-to-gel transition temperature by placing 2 mL of each gel individually in a test tube, which was placed in a thermostatic water bath (oscillating thermostatically controlled shaker, GallenKamp, England), with each tube covered with Parafilm^®^. The temperature of the water bath was gradually raised from 25 °C to 37 °C in increments of 3 °C with a sufficient time (15 min) to equilibrate at each new temperature setting [47]. The sample gelation temperature was identified when the meniscus does not move by tilting the test tube with a 90° angle [48,49].

The sol-to-gel transition time was detected by the test tube inversion technique by transferring 1 mL of the formulated gels in a stoppered test tube and then placing the tubes in a thermostatically controlled water bath adjusted at 37 °C. The sol-to-gel transition time was estimated when the test tube was tilted every 10 s and no flow was noticed [50].

##### Assessment of Rheological Properties

Temperature sweep test was used for the assessment of temperature-dependent complex viscosity [51]. The rheological properties were assessed using viscoelastometer (Anton Paar, MCR301 SN80218500, Austria) with a parallel plate geometry (gap of 0.5 mm), a temperature control system (Peltier system), and Rheoplus/32 V3.40 software version (Anton Paar, Austria). This was carried out by placing the gel sample (5 mL) in a shearing jig and testing them at a frequency of 1 Hz and amplitude gamma of 1%. A gradual increase in temperature from 25 to 37 °C was made with an elevation rate of 2 °C/min [52]. Complex viscosity (ɳ^*^) was plotted against temperature (T) to detect the ability of the tested samples to transform into a gel at the physiological temperature, thus aiding in the choice of the optimum in situ gel formulation.

##### In Vitro Drug Release

Dialysis cellulose bag method was adopted for the assessment of drug release parameters. The in vitro drug release was carried out for the optimum RSM-loaded transferosomal formulation along with the formulated in situ gels loaded with the optimized transferosomal formulation. In brief, equivalent volumes of each sample (corresponding to 1 mg drug) were placed in a dialysis cellulose bag, which was pre-soaked overnight in distilled water. The packed cellulose bags were tightly secured from both ends to avoid any leakage. The secured bag was placed in a glass bottle pre-filled with 20 mL ethanolic PBS solution (10% *v*/*v*, pH 7.4) [53,54,55] then transferred to a thermostatically controlled shaker adjusted at 100 rpm at 37 °C ± 0.5 [56]. Aliquots (2 mL) of the release medium were withdrawn at different pre-determined time intervals (0.5, 1, 2, 3, 4, 5, 6, and 8 h) and instantly replaced by equal volume of freshly prepared release medium. The collected samples were then evaluated spectrophotometrically at λ_max_ 271 against blanks. The measured release parameters were the % drug released after 0.5 h (Q_0.5h_) to assess the initial drug flush release, in addition to the % of drug released after 8 h (Q_8h_) to ensure the sustainability of drug release upon incorporation in gel formulations. The same aforementioned procedure (without blank) was carried to assess the release of the pure drug from its dispersion for comparison purposes. The % of drug released was illustrated against time.

#### 2.2.6. Effect of Storage

The effect of storage of the optimized RSM-loaded transferosomal formulation along with the respective optimum in situ gel containing the optimum transferosomal formulation was assessed at 4 ± 2 °C for three months [57,58]. At the end of the study period, evaluation of VS, PDI, ZP, and %EE was performed for the optimized transferosomal formulation in addition to the sol-to-gel transition temperature re-evaluation for the selected in situ gel containing the optimized RSM-loaded transferosomal formulation. In vitro drug release was performed for both the optimum transferosomal formulation as well as the optimum in situ gel formulation loaded with it. These procedures were held along with visual inspection for any physical changes, sedimentation, or aggregation [57]. The similarities between the release profiles of the freshly prepared optimum transferosomal formulation and the stored one, as well as, the optimized fresh in situ gel formulation and the stored gel, were calculated using the model-independent mathematical approach proposed by Moore and Flanner [59].

(2) $${ƒ}_{2}=50.\log\;[{\left\{1+\left(\frac{1}{n}\right)\sum_{t-1}^{n}{\left(R_{t\;}-T_{t}\right)}^{2}\right\}}^{-0.5}\times 100]$$

where n is the number of the samples withdrawn, and R and T are the percentage of RSM released from either the freshly prepared or stored formulation (F12), respectively, which is repeated for the freshly prepared and stored in situ gel formulation (G2) at time t [60,61].

#### 2.2.7. In Vivo Studies

The in vivo animal studies were performed on male Wistar Albino rats weighing 200–250 g. Animal use was in agreement with the National Institutes of Health Guide for The Care and Use of Laboratory Animals (NIH, publication No.85–23, 1996). The study protocol was accepted by the institutional review board, Research Ethics Committee, Faculty of Pharmacy, Cairo University [PI (3043)]. The rats were housed in cages, with each containing six animals, at ambient temperature and pressure, with free entry of food and water, and a 12 h light and dark cycle.

##### Assessment of Formulation Biocompatibility

In order to assess the safety and the biocompatibility of the applied in situ gel formulation on the nasal epithelial cell membrane, histopathological evaluation was carried out after applying the optimum RSM-loaded transferosomal in situ gel (4 mg/kg) intranasally [62] to four male Wistar Albino rats. On the other hand, another four rats were used as control and did not receive the in situ gel. Four hours post application, the rats were mercy sacrificed, then the nasal epithelial cell membrane was dissected to be examined for any harmful effects [25,46]. Specimens were fixed in 10% neutral buffer formalin, then decalcified by 10% formic acid, trimmed, washed in water, dehydrated in ascending grade of ethyl alcohol, cleared in xylene, and lastly fixed in paraffin at 70 °C before being prepared in paraffin blocks. Thin sections (4–6 µm) were processed using microtome and stained with hematoxylin and eosin before being inspected by Olympus BX53 microscope [63].

##### In Vivo Pharmacokinetics

The study was performed so as to determine the pharmacokinetic parameters of the intranasally administered RSM-loaded transferosomal in situ gel (G2) compared with the intravenous (IV) RSM aqueous solution, which was administered to male Wistar Albino rats. A non-blind, one-period, randomized parallel design was applied. Sixty-six Wistar Albino rats were selected for the study. The animals were distributed over two equal groups; Group 1 received IV RSM aqueous solution and Group 2 received IN RSM-loaded transferosomal in situ gel (G2). The dose of RSM in both treatments was corresponding to 4 mg/kg drug [62]. The study was carried out over 12 h and the animals were visually examined along the study period to detect any behavioral disorder or illness. At time intervals 0 (pre-dose), 0.25, 0.5, 0.75, 1, 1.25, 1.5, 2, 4, 6, 8 and 12 h post administration of each treatment, three rats from each group were mercy sacrificed for the collection of respective plasma and brain samples. Blood samples were taken from the trunk of the sacrificed animals directly into heparinized tubes followed by centrifugation (Beckman, Fullerton, Canada) at 4000 rpm for 15 min at 25 °C to separate plasma [64], while brain tissue samples were collected by opening the sacrificed animal’s skull, dissecting the brain, then removing any adhering tissues followed by homogenization with saline (three-fold its volume) using ultra Turrax homogenizer (T 25 digital ULTRA- TURRAX^®^, IKA, Germany) at 24,000 rpm for 1 min [65]. Both plasma and brain samples were maintained at −20 °C pending analysis.

##### Chromatographic Conditions

The assessment of RSM in plasma and brain tissues was carried out applying a sensitive, selective, and validated LC-MS/MS [66]. The internal standard used was clonazepam. The detector used was Triple Quarupole MS/MS (Waters Corp., Milford, MA, USA). Analyst 1.6.3 software version was utilized for data acquisition and integration. The used column was a reverse-phase column (C_18_, 4.6 × 50 mm, 5 um, Waters Corp., Milford, MA, USA). The mobile phase was prepared by mixing calcium ammonium nitrate and 0.01 M ammonium formate in the ratio 4:1 (*v*/*v*). Isocratic chromatographic separation was carried out at 15 °C with a flow rate of 1 mL/min.

##### Sample Preparation

Liquid–liquid extraction was performed by adding 0.5 mL of either plasma or homogenized brain samples to 4 mL ethyl acetate and vortexing for 30 s, then centrifuging at 3000 rpm at 4 °C for 15 min. The organic layer was evaporated until dryness under a gentle nitrogen stream at 40 °C (Vacufuge 5301, Eppendorf, Hamburg, Germany), and the residue was then reconstituted with 100 µL mobile phase and vortexed for 1 min. A volume of 20 µL aliquot of the reconstituted sample was injected into the column for analysis.

##### Brain and Systemic Kinetic Analysis

The pharmacokinetic parameters in both plasma and brain tissue homogenates were calculated for comparative studies between IN and IV administration. For investigated plasma samples, the mean RSM plasma concentration was plotted versus time in a plasma concentration-time curve. Kinetica^®^ software (version 5.0, 2017, Thermo Fisher Scientific, Waltham, MA, USA) was used for the calculation of peak plasma concentration (C_max_), the time required to reach it (T_max_) along with the area under plasma concentration-time curve over 12 h (AUC_0-12_). Absolute bioavailability of RSM in plasma after IN administration was calculated relative to the IV RSM aqueous solution.

For brain samples, the mean RSM brain concentration was plotted versus time, and the pharmacokinetic parameters (C_max_, T_max_, and AUC_0-12_) were determined following the same way previously mentioned for plasma samples. RSM bioavailability in brain tissues from IN administration was calculated relative to the IV RSM aqueous solution.

To follow RSM targeting to the brain, drug targeting efficiency (DTE) and nose-to-brain direct transport percentage (DTP) [67] were calculated as follows:

(3) $$DTE\left(\%\right)=\left(\;\frac{{\left(\frac{B}{P}\right)}_{IN}}{{\left(\frac{B}{P}\right)}_{IV}}\;\right)\times 100$$

(4) $$DTP\;\left(\%\right)=\left(\;\frac{B_{\mathrm{IN}}-B_{X}}{B_{\mathrm{IN}}}\;\right)\times 100$$

where B and P stand for AUC_0-12_ in brain and plasma, correspondingly. IN and IV represent intranasal and intravenous administration, correspondingly, and B_*X*_ is the portion of the drug that reached the brain from blood–brain barrier (BBB) following IN administration. The following equation was used for the calculation of B_*X*_ [67],

(5) $$B_{X}=\left(\;\frac{B_{\mathrm{IV}}}{P_{\mathrm{IV}}}\;\right)\times P_{\mathrm{IN}}$$

##### Statistical Analysis

To statistically compare different formulations, either Student’s *t*-test or analysis of variance (ANOVA) was conducted using Microsoft excel. Significant differences are determined when *p* values are < 0.05.

## 3. Results and Discussion

### 3.1. Characterization of RSM-Loaded Transferosomes

#### 3.1.1. Assessment of Vesicle Size (VS), Polydispersity Index (PDI), and Zeta Potential (ZP)

**Figure 1:**
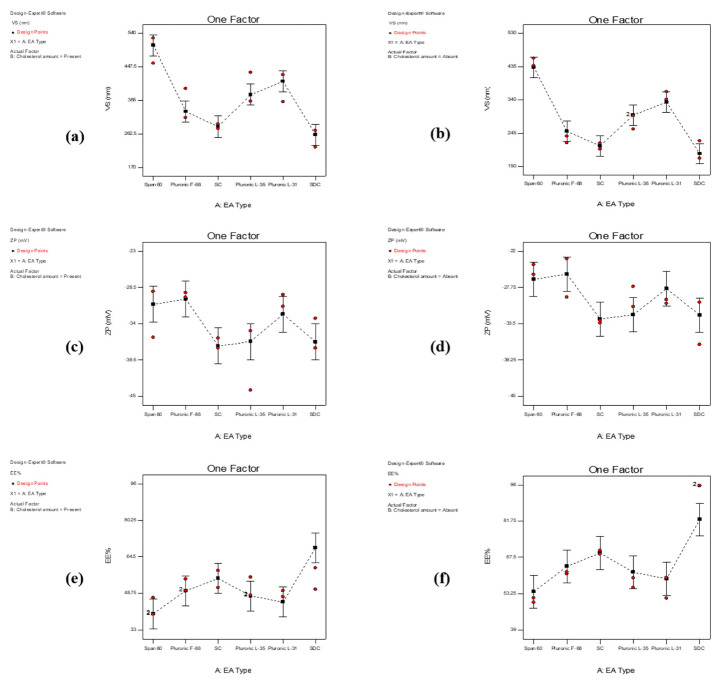
Line plot for the effect of EA on VS (**a**) with cholesterol, (**b**) without cholesterol; on ZP (**c**), with cholesterol, (**d**) without cholesterol; and on %EE (**e**) with cholesterol, (**f**) without cholesterol.

According to Table 2, and as illustrated in Figure 1a,b, it could be demonstrated that the prepared transferosomal formulations showed VS values ranging from 198.63 ± 34.98 to 491.40 ± 48.79 nm. As illustrated in the figure, it is obvious that both the type of EA and the presence of cholesterol had a significant influence on the VS (*p* < 0.0001 and *p* = 0.0001, respectively). Regarding the type of the EA, the VS was greatly affected by either the HLB value, ionic nature, or the molecular structure of the used EA. The behavior of anionic EA was different from that of nonionic EA. Upon comparing the VS of the formulations prepared using nonionic EA, we found a significant reduction in the VS with the increase in the HLB value of the used EA. The used nonionic EA can be descendingly ordered according to their HLB values as follows: Pluronic^®^ F-68 > Pluronic^®^ L-35 > Pluronic^®^ L-31 > Span^®^ 60. The obtained VS values for the transferosomes prepared using the previously mentioned EA were 227.51 ± 13.69, 276.00 ± 27.05, 352.90 ± 15.41, and 448.70 ± 14.84 nm, respectively, in absence of cholesterol. This enhanced decrease in the obtained VS values might be attributed to the decrease in interfacial tension during vesicle formation using higher HLB values [68]. On the other hand, the obtained VS values of the formulations prepared by anionic EA (sodium cholate and sodium deoxycholate) were the smallest (208.42 ± 11.95 and 198.63 ± 34.98 nm, respectively) compared with those prepared using the nonionic EA. This enhanced decrease in the VS might be due to the steric repulsion between the charged EA molecules arranged on the surface of the vesicles causing an increase in the curvature of the vesicles membrane [69], in addition to the insignificant difference between the VS of the transferosomes prepared using sodium cholate and sodium deoxycholate owing to the nearly similar HLB values (18 and 16, correspondingly) as well as the molecular weight values (430.6 and 414.6, respectively).

Conversely, the presence of cholesterol had a significant influence on increasing the VS (*p* = 0.0001) of the formed transferosomes compared with those lacking its addition. This might be attributed to the bulky structure of cholesterol, which led to an increase in the VS of the formed vesicles [70]. The incorporation of cholesterol in the lipid bilayer might have resulted in more aqueous distribution within the vesicle due to the interference with the closed packing of the lipid bilayer [71,72]. Design expert plots representing the influence of the studied factors are demonstrated in Figure 1a,b.

PDI is an assessment of size distribution that affects the homogeneity of the tested samples. It was previously reported that PDI values < 0.7 indicate narrow vesicle size distribution [73,74]. Since PDI values ranged from 0.3 ± 0.02 to 0.68 ± 0.02, this indicated the narrow size distribution and homogenous dispersion of the formed vesicles and, hence, their better stability.

It has been reported that formulations with ZP values exceeding +30 mV or −30 mV are said to be more stable with less aggregation probability [75,76]. As demonstrated in Table 2 and Figure 1c,d, the obtained ZP values ranged from −39.60 ± 6.36 to −24.90 ± 1.13 mV. Both the type of EA and the presence of cholesterol imparted a significant effect (*p* = 0.0219 and *p* = 0.0038) on the ZP value. Regarding the type of EA, it was previously reported that anionic EA, being negatively charged, possesses a significant effect on ZP at an EA concentration of 1 g/L [77], and since the used concentration of EA was 3.33 g/L, it obviously led to the enhancement of the negative value of ZP. On the other side, it was deduced that the presence of cholesterol increased the negative value of ZP, which might be due to the effect of cholesterol on the arrangement of EA molecules at the interface of the vesicles [78].

#### 3.1.2. Assessment of Entrapment Efficiency (%EE)

As demonstrated in Table 2 and Figure 1e,f, the values of EE ranged from 43.25 ± 5.02 to 95.73 ± 0.09. It was shown that the type of EA affected the %EE significantly (*p* = 0.002) in the absence of cholesterol, while it imparted a non-significant difference in its presence. The ionic nature, the molecular size, and the HLB values of the used EA played a vital role in entrapping RSM effectively. Regarding the ionic nature of the used EA, it was obvious that using the anionic EA, sodium cholate, or deoxycholate showed higher EE values relative to those prepared using nonionic ones [70], which might be due to the protonation of the amino group in the propargylamine residue of the drug. This protonated amino group may have formed an electrostatic bond with the negative charge of the anionic EA [79]. Additionally, upon comparing both the anionic EAs used, it was found that the highest EE values were obtained for the vesicles formed using sodium deoxycholate. This could be attributed to the small molecular size (414.6 g/mol) of sodium deoxycholate [80,81] resulting in a less steric hindrance upon closure of the vesicles [68]. With regard to the transferosomal formulations prepared using nonionic EA, we found that the %EE increased ascendingly with the respective increase in the HLB values. The lowest and highest %EE values were noted for transferosomes formed using Span^®^ 60 (HLB = 4.7) and Pluronic^®^ F-68 (HLB = 29), respectively. This was in accordance to a previous study that encapsulated the hydrophilic drug N-acetyl glucosamine within niosomes. EA with higher HLB yielded vesicles with the larger aqueous spaces needed for drug entrapment within the formed lamellae and provided greater protection for drug leakage from the formed niosomes, resulting in higher encapsulation efficiency [82].

Additionally, the statistical analysis of the obtained results gave a significant effect (*p* = 0.0005) of the presence of cholesterol on the %EE values. It was obvious that adding cholesterol significantly decreased the %EE values for the prepared transferosomal formulations (*p* = 0.0005). This might be due to the steric hindrance caused by cholesterol, which limited the drug inclusion in the formed vesicles [83]. On the other hand, it is believed that the addition of cholesterol led to disruption of the linear structure of transferosomes, causing a negative impact on %EE values [70]. Furthermore, the addition of cholesterol might result in increasing the bilayer interfacial region hydrophobicity, resulting in less entrapment for the drug [84].

### 3.2. Optimization of the Formulation Variables

One formulation combining the desirable characteristics (with desirability value = 0.749) (F12, containing sodium deoxycholate as EA in the absence of cholesterol) was selected as the optimized RSM-loaded transferosomal formulation. The optimum selection of different variables was acquired by numerical optimization based on the desirability value obtained from Design-Expert^®^ software [85]. F12 demonstrated the following values (VS: 198.635 ± 34.98 nm, PDI: 0.45 ± 0.079, ZP: −33.45 ± 4.73 mV, and %EE: 95.735 ± 0.091%). Additional characterization tests regarding its morphology and the compatibility of the drug with other used components are demonstrated below. Moreover, it was further incorporated within in situ gel formulations.

### 3.3. Characterization of the Optimized Transferosomal Formulation (F12)

#### 3.3.1. Fourier Transform Infrared Spectroscopy (FTIR)

**Figure 2:**
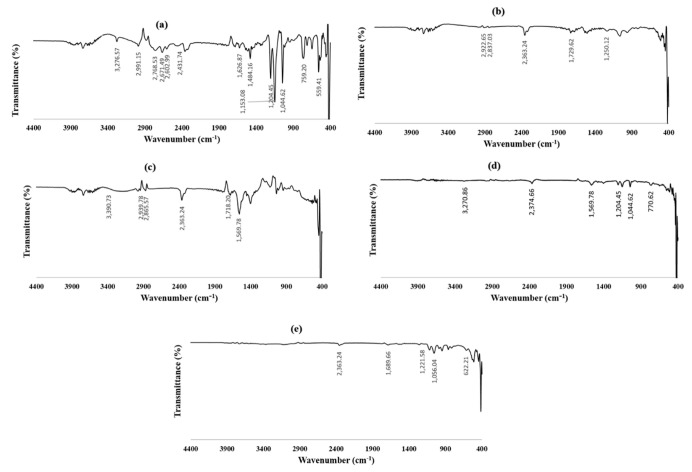
FTIR spectra of (**a**) RSM, (**b**) phosphatidylcholine, (**c**) sodium deoxycholate, (**d**) physical mixture of the components, and (**e**) optimized RSM-loaded transferosomes (F12).

As shown in Figure 2a, the FTIR spectrum for pure RSM revealed characteristic sharp peaks at 3276.57 cm^−1^, 1626.87 cm^−1^, 1204.45 cm^−1^, 1044.62 cm^−1^, and 759.2 cm^−1^ owing to propargyl group ≡C–H stretching vibration, the stretching of N– H...O, symmetric and asymmetric S–O stretching, and stretching of the C–S bond, respectively.

Figure 2b shows the FTIR spectrum of phosphatidylcholine with significant peaks at 2922.65 cm^−1^ and 2837.03 cm^−1^ due to long-chain fatty acid C-H stretching [86]. Symmetric C=O stretching vibration was observed at 1729.62 cm^−1^ [87], and peaks of phosphate-groups-building phospholipid appeared at 1250.12 cm^−1^ [88].

FTIR absorption peaks for sodium deoxycholate were illustrated in Figure 2c, showing the broad band of –OH stretching vibrations that appeared at 3390.73 cm^−1^. At 2939.78.49 and 2865.57 cm^−1^, two bands were detected, which represented symmetric and asymmetric -CH_2_ stretching vibrations, respectively. A carboxylate ester group C=O stretching vibration band was detected at 1569.78 cm^−1^ [89].

Regarding the FTIR spectra of the physical mixture (Figure 2d), the peak intensity at 3276.57 cm^−1^ significantly decreased, while the characteristic peaks of RSM were still present with the preservation of the other components peaks. On the other hand, in the optimum formulation illustrated in Figure 2e, the peak at 3276.57 cm^−1^ completely disappeared, which might be due to the inclusion of the drug inside the formed vesicles. This is in addition to the absence of the peak sharpness of the other peaks, indicating the drug incorporation in the formed transferosomes. The shifting of some peaks from 1626.87, 1044.62, and 1204.45 cm^−1^ to 1689.66, 1056.04, and 1221.58 cm^−1^ may be due to a dilution effect, which represents the achievement of drug entrapment inside the vesicles while retaining its properties [53,90]. This might indicate the absence of interaction between the components of the formulation [91].

#### 3.3.2. Transmission Electron Microscopy (TEM)

**Figure 3:**
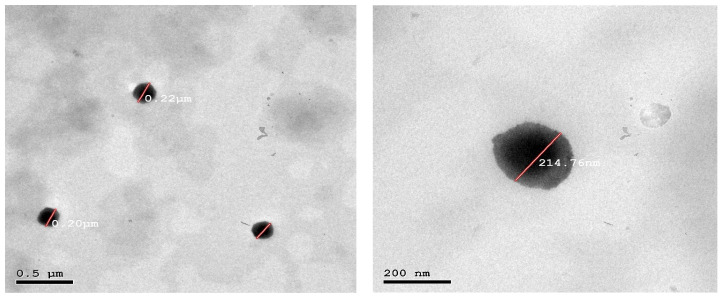
TEM micrographs of the optimized RSM-loaded transferosomes (F12).

TEM images of the optimized RSM-loaded transferosomal formulation (F12) are illustrated in Figure 3. It is obvious that the transferosomal vesicles were well-identified and spherical in shape with nearly perfect edges. The measured VS was in good correlation (~200 nm) with the results previously obtained from ZetaSizer Nano ZS.

### 3.4. Characterization of Mucoadhesive RSM-Loaded Transferosomal In Situ Gels

The in situ gel is a type of pharmaceutical formulation that is liquid (sol) outside the human body and transforms into a gel consistency inside the human body under physiological conditions [24]. The in situ gel prepared in the current study depended on the use of a mixture of thermosensitive polymers, such as Pluronic^®^ F-127 and Pluronic^®^ F-68, in addition to a mucoadhesive agent, such as pectin, in different concentrations.

Pectin is a natural polysaccharide capable of forming a three-dimensional network in the gel structure [92]. Optimum concentrations selection is based on the sol-to-gel transition temperatures and gelation time [30].

In this study, various concentrations of Pluronic^®^ F-68 were added to a single concentration of both Pluronic^®^ F-127, in addition to the mucoadhesive polymer, pectin (0.5% *w*/*v*), to prepare three in situ gels (G1, G2, and G3) containing the optimized RSM-loaded transferosomal formulation (F12) (Table 3). The cold method adopted produced homogenous yellowish dispersions that were visually inspected with no aggregates in liquid form at ambient temperature. The formulated RSM-loaded transferosomal in situ gels were assessed for their sol-to-gel transition temperature, rheological properties, and in vitro drug release for the selection of the optimum in situ gel.

#### 3.4.1. Sol-to-Gel Transition Temperature

As shown in Table 3, it was obvious that the sol-to-gel temperature was up to 40.1 °C. By analyzing the results, it was manifested that the incorporation of Pluronic^®^ F-68 in the composition of the in situ gel (prepared using single concentration of Pluronic^®^ F-127; 15% *w*/*v*) significantly elevated (*p* < 0.05) the sol-to-gel transition temperature. Moreover, it was noticed that elevating the concentration of Pluronic^®^ F-68 from 5 to 10% *w*/*v* led to a corresponding increase in the sol-to-gel transition temperature from 36.2 ± 0.282 °C to 40.1 ± 1.69 °C. This observation might be attributed to the presence of a higher PEO/PPO ratio in the structure of Pluronic^®^ F-68 compared with Pluronic^®^ F-127 [52]. As previously reported by Yuan et al., who explained the idea that Pluronics with high hydrophobic PPO (such as Pluronic^®^ F-127) proportion had low sol-to-gel transition temperature, while those composed of high hydrophilic PEO (such as Pluronic^®^ F-68) proportion demonstrated higher gelation temperatures [93]. This confirms the result that the incorporation of Pluronic^®^ F-68 having higher PEO/PPO ratio compared with Pluronic^®^ F-127 led to the elevation of gelation temperature [52]. By checking the results of the sol-to-gel transition time, it was obvious that the obtained values were around 15 s.

#### 3.4.2. Rheological Study

**Figure 4:**
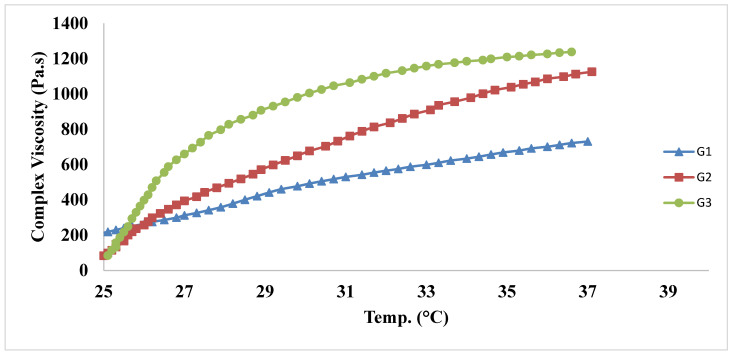
Relationship between temperature and complex viscosity of in situ gels: G1, G2, and G3. Composition of the in situ gels is compiled in Table 3.

The rheological properties assessment illustrated in Figure 4 confirmed that upon increasing the concentration of Pluronic^®^ F-68, a relative increase in the complex viscosity occurred. The elevation of the complex viscosity was in the order that G1 (lacking Pluronic^®^ F-68) was the first to form the gel, followed by G2 (5% *w*/*v* Pluronic^®^ F-68) and then G3 (10% *w*/*v* Pluronic^®^ F-68). The estimated complex viscosity values were 128.0, 99.3 and 84.2 Pa.s at 25 °C for G1, G2, and G3, while they reached 730, 1130, and 1240 Pa.s, respectively, at 37 °C. The results indicated the increased complex viscosity upon elevating the concentration of Pluronic^®^ F-68 (G1 < G2 < G3).

#### 3.4.3. In Vitro Drug Release

**Figure 5:**
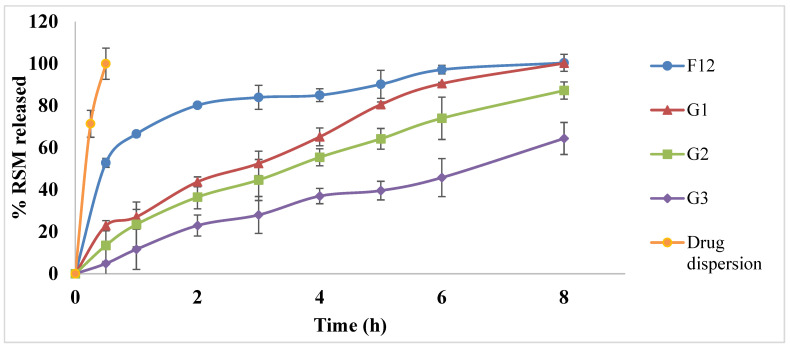
Release profiles of RSM from optimized RSM-loaded transferosomal formulation (F12) and prepared in situ gels (G1, G2, G3) in ethanolic phosphate-buffered saline; pH 7.4. Composition of the in situ gels is illustrated in Table 3.

It was detected that the drug was totally released from the prepared drug dispersion in 0.5 h. Figure 5 represents the percentage of RSM released from the optimized RSM-loaded transferosomal formulation (F12) and the prepared in situ gels (G1, G2, and G3) in ethanolic PBS solution (10% v/v, pH 7.4) in addition to the drug dispersion. From the figure, it is obvious that the optimized transferosomal formulation F12 suffered from a flush release of 52.79 ± 2.1% within the first 0.5 h (Q _0.5 h_), followed by a slower release where the % of drug released after 8 h (Q_8h_) was 100.42 ± 4.02%. This initial flush might be due to the release of the drug entrapped near the surface layers of the formed transferosomes, while the rest of the drug entrapped in the deep layers was released in a slower manner later [94]. On the other hand, the incorporation of F12 within the in situ gels controlled the initial burst release of RSM, where the Q_0.5h_ values were 4.86 ± 6.87, 13.50 ± 7.64, and 22.89 ± 2.39% for G3, G2, and G1, respectively, with a significant reduction (*p* < 0.05) when compared with the free transferosomal formulation (F12). This might be due to the enhanced viscosity of the formed gels, which in turn reduced the drug release [95]. Q_8h_ values were assessed to assure the sustainability of drug release in gel formulations and evaluate the effect of Pluronic^®^ F-68 on the gelation temperature, so by comparing the Q_8h_ values of the investigated in situ gels containing the optimized formulation, F12, it was evident that the obtained values were significantly smaller than that of the free transferosomal formulation. The obtained Q_8h_ values were 100.25 ± 0.62, 87.25 ± 4.11, and 64.42 ± 7.63% for G1, G2, and G3, respectively. Furthermore, it is obvious that the presence of Pluronic^®^ F-68 and increasing its concentration led to a significant reduction in the % of drug released after 0.5 and 8 h. This might be due to the increased viscosity of the gel at 37 °C due to the incorporation of Pluronic^®^ F-68, which is in accordance with Din et al. [96].

From the preceding findings, it can be deduced that the in situ gel (G2) was the best regarding its sol-to-gel transition temperature of 36.2 ± 0.282°C, as well as its controlled release behavior for RSM. The selected in situ gel formulation (G2) was investigated for further studies.

### 3.5. Effect of Storage

**Figure 6:**
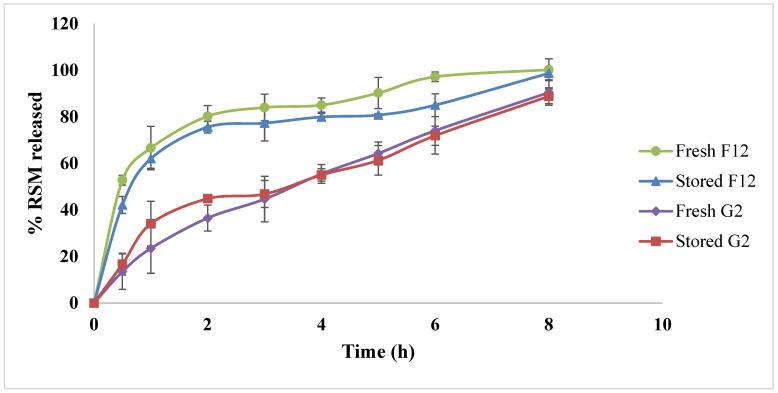
Release profiles of RSM from fresh optimized RSM-loaded transferosomal formulation (F12), stored (F12), fresh in situ gel (G2), stored (G2) in ethanolic phosphate-buffered saline; pH 7.4.

The optimized RSM-loaded transferosomal formulation (F12) along with the respective optimum in situ gel (G2) were re-evaluated after storage for three months at 4 °C. All refrigerated gel formulations showed no sedimentation or aggregations. The stored free transferosomal formulation, F12, was re-evaluated for its VS, PDI, ZP, and %EE measurements, and the results revealed that the obtained values (294.85 ± 20.29 nm, 0.505 ± 0.021, −37.85 ± 0.77 mV, and 89.55 ± 1.62%, respectively) were insignificantly different compared with the freshly prepared formulation (*p* > 0.05). Moreover, the stored in situ gel (G2) showed an insignificant difference (*p* > 0.05) regarding the sol-to-gel transition temperature when compared with the freshly formed one, where the obtained values were 36.81 ± 0.47 versus 36.2 ± 0.282, respectively. In vitro RSM release profiles revealed an insignificant difference (*p* > 0.05) between the freshly prepared transferosomal formulation (F12) and the stored one, as well as the freshly prepared in situ gel (G2) and the stored one, as shown in Figure 6. Furthermore, to be approved by the FDA guidance for industry, the similarity factor should be above 50 in order to ensure the similarity between the dissolution profiles [59]. Since the similarity factor for the optimum formulation F12 before and after storage was 54, and that of the in situ gel formulation (G2) before and after storage was 64, the physical stability of the prepared transferosomal formulation and the corresponding in situ gel could be confirmed, with no drug leakage from the formed vesicles.

Composition of the in situ gels is compiled in Table 3.

### 3.6. In Vivo Studies

All the animals subjected to in vivo studies tolerated the drug and were alive with the same level of vitality, showing normal behavior along the whole sampling time.

#### 3.6.1. Assessment of Formulation Biocompatibility

**Figure 7:**
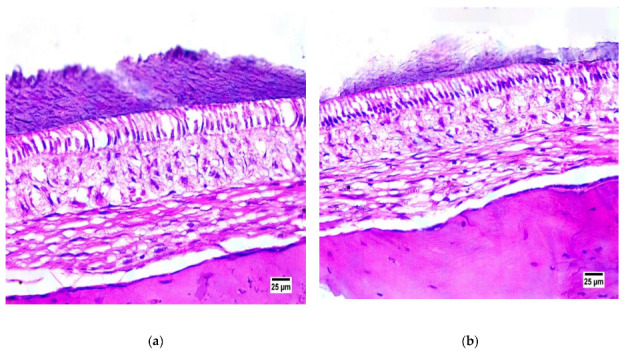
Photomicrographs showing normal histological structure of nasal mucosa in (**a**) control group (untreated) and (**b**) intranasally administered RSM-loaded transferosomal in situ-gel-treated group (stain used is hematoxylin and eosin).

To detect the biocompatibility and safety of the administered IN in situ gel containing the optimum RSM-loaded transferosomal formulation (G2), a histopathological study was performed on male Wistar Albino rats. As illustrated in Figure 7, it is obvious that no significant changes could be detected in the treated group compared with the untreated control group, indicating the absence of any damaging effects on the microscopic structure of the nasal mucosa. In the treated group the nasal mucosa of the sacrificed rats remained intact with no severe signs of necrosis or hemorrhage, furthermore, the epithelial layer was intact with no change in the basal membrane and the superficial part of the submucosa. The obtained results might be attributed to the use of safe components for the formulation of the in situ gel. Several researchers studied the safety of the drug [97], as well as the used transferosomal components, such as phosphatidylcholine [36] and sodium deoxycholate [98], and the in situ-forming gel components, such as Pluronics and pectin [99,100], for IN delivery. Thus, these results indicate that RSM-loaded transferosomal in situ gel (G2) is considered as a safe and biocompatible formulation for IN administration.

#### 3.6.2. In Vivo Pharmacokinetics

The calibration curve of the conducted LC-MS/MS technique presented a lower limit of quantification of 0.1 ng/mL in both plasma and brain tissues, with good linearity from 0.1 to 1000 ng/mL (R^2^ of the calibration curve lines were 0.9995 and 0.9993 for plasma and brain, respectively).

**Figure 8:**
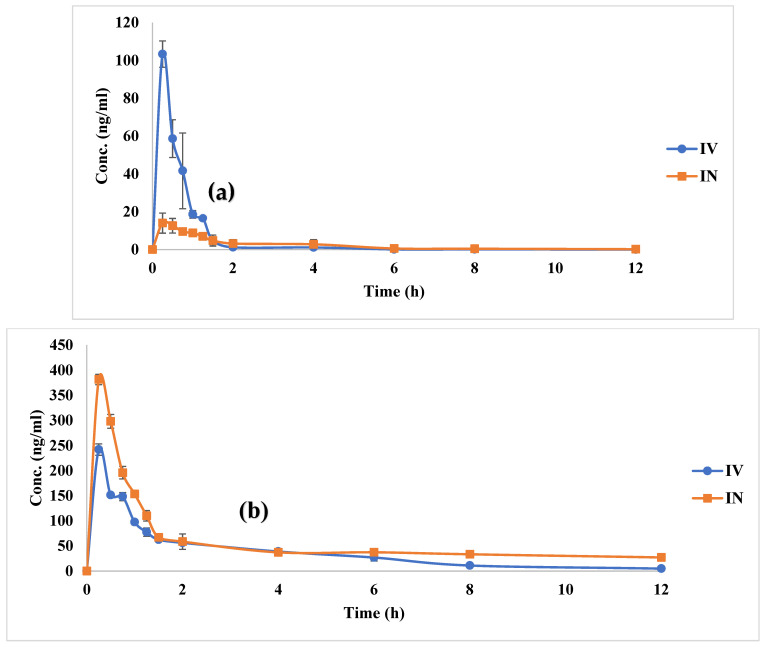
RSM concentration-time curve following the administration of RSM IV aqueous solution and RSM IN in situ gel (G2) in (**a**) plasma and (**b**) brain tissues.

RSM concentrations in plasma and brain homogenates were illustrated against time, and the constructed curves were illustrated in Figure 8a,b. The calculated pharmacokinetic parameters, C_max_, T_max_, and AUC_0-12_, are represented in Table 4.

From the RSM plasma concentration-time curve (Figure 8a), it is obvious that both the investigated samples, RSM IV aqueous solution and RSM-loaded transferosomal in situ gel (G2), possessed different shapes. It is clear that the plasma concentration-time curve of the IV sample possessed higher C_max_ than the IN-administered in situ gel formulation. Moreover, comparable T_max_ could be detected. Table 4 shows the pharmacokinetic parameters of both samples in the plasma. From the table, it is found that the obtained C_max_ values were 103.30 ± 6.95 and 14.01 ± 5.30 ng/mL after 0.25 h (T_max_) following IV and IN administration, respectively. From the obtained values, we observed that the C_max_ value of IN-administered in situ gel (G2) was significantly lower (*p* = 0.0012) than that of IV aqueous solution. Alternatively, there was non-significant difference in T_max_ as C_max_ was reached after 0.25 h for both groups. Concerning the AUC_0-12h_, it was clear that the calculated values for IN in situ gel (27.31 ± 5.60 ng.h/mL) was significantly less (*p* = 0.001) than that of IV aqueous solution (63.4 ± 3.00 ng.h/mL), which might explicate the drug availability in brain tissues rather than plasma [101]. The absolute bioavailability of the drug in plasma after IN in situ gel application was calculated to be 43.07%. This value being less than 100% might indicate the brain targeting of the drug. Further confirmations on the efficient brain targeting was indicated after the calculation of RSM concentration in the brain tissues.

By investigating the RSM concentration in the brain tissues and studying the brain concentration-time curve (Figure 8b), it is manifested that the IV aqueous solution, as well as the IN-administered in situ gel, showed differently shaped curves, where the IN-administered formulation possessed higher C_max_ and comparable T_max_ values compared with the IV aqueous solution. By comparing the calculated results of the brain kinetics parameters (Table 4), it is observed that C_max_ following IN in situ gel administration was significantly higher (*p* = 0.0014) than that following IV aqueous solution, where the obtained values were 381.58 ± 10.15 and 241.93 ± 11.41 ng/mL after 0.25 h (T_max_) for the aforementioned treatments, respectively. From the table, the calculated AUC_0-12h_ values showed significant differences (*p* = 0.002) between the IN-administered in situ gel and IV aqueous solution, where the obtained values were 586.47 ± 31.50 versus 447.08 ± 31.49 ng.h/mL for the previously mentioned treatments, respectively. The brain bioavailability of the drug from IN in situ gel was 131.17% relative to the IV aqueous solution, which could indicate that nasal application of the formulated IN in situ gel succeeded in the brain targeting of the drug.

To confirm the brain targetability of the optimized in situ gel formulation, drug targeting efficiency (DTE%) and direct transport percentage (DTP%) were used for comparing the AUC_0-12h_ values obtained from the drug concentration-time curve in plasma and homogenized brain tissues. It is believed that a value of DTE% equal to 100% means that the drug concentration is in equal proportion in the brain following both IV aqueous solution and IN in situ gel administration. On the other hand, if the value of DTE% > 100%, then the drug is preferentially targeted to the brain following IN administration, and if DTE% < 100%, then the drug is preferentially targeted to the brain following IV administration [102]. Calculated DTE% was found to be 304.53%, confirming that IN administration of the in situ gel led to a better brain targeting. This might be due to the incorporation of pectin as a mucoadhesive polymer that increased the residence time in the nasal mucosa, in addition to the use of Pluronic^®^ F-68 that might have increased the complex viscosity and gelation temperature, which was high enough to transform into gel at physiological temperature as previously mentioned, which in turn increased the AUC_0–12 h_.

The other parameter of the drug, namely DTP%, was assessed in order to determine whether the drug reached the brain through the BBB or directly from the nose-to-brain through the olfactory pathway [102]. The calculated DTP% of the tested IN in situ gel was 67.16%, suggesting the predominance of direct nose-to-brain transport following the IN in situ gel administration. The high DTE% and DTP% values confirmed the successful brain targeting of RSM.

By comparing the values of the calculated DTE% and DTP% for the developed in situ gel containing RSM-loaded transferosomes with other studies in the literature designed for the brain targeting of the drug, it is observed that the developed formulation in the current study succeeded in enhancing the brain targeting. One study tested the brain targeting of the RSM-loaded chitosan glutamate nanoparticles after intranasal administration, and the results revealed that the DTP% was calculated to be 69.27 ± 2.1% [3]. Another study addressed the preparation of RSM-loaded gellan gum transdermal patches, where the obtained DTE% and DTP% values were 193.75% and 48.15%, respectively [12].

The obtained results in the current study and the efficient brain targeting could be ascribed to the small VS of the formed transferosomes [103]. Furthermore, the Pluronic^®^ F-127 in the formed in situ gel might have resulted in entanglement with the glycoprotein chains of the nasal mucosa, which can permit longer residence and, hence, absorption [104,105]. Finally, the presence of pectin as mucoadhesive polymer assured the reduced drug mucociliary clearance and increased the residence of the formulation onto the nasal mucosa.

## 4. Conclusions

In the present study, in situ-forming gels containing rasagiline mesylate-loaded transferosomes were successfully prepared for efficient intranasal delivery of the drug as a way to bypass the first-pass metabolism. Among the prepared transferosomal formulations and after statistical analysis of the results, it was found that the transferosomal formulation (F12) prepared using sodium deoxycholate as an EA and lacking the addition of cholesterol possessed the lowest vesicle size < 200 nm and highest entrapment efficiency value > 95%. The optimum transferosomal formulation was loaded into an in situ-forming gel prepared using a mixture of Pluronics (15% *w*/*v* Pluronic^®^ F-127 & 5% *w*/*v* Pluronic^®^ F-68) along with a mucoadhesive polymer—0.5% pectin. In vitro drug release studies revealed the significant effect of the prepared in situ gel on reducing the initial burst obtained from the transferosomal dispersion. The selected in situ gel possessed excellent cytocompatibility on the nasal mucosa of rats. Furthermore, a significant enhancement in the brain delivery of the drug was obtained after the intranasal delivery of the gel formulation compared with the intravenous delivery of RSM aqueous solution, where the brain bioavailability was calculated to be 131.17%. The obtained results indicated the development of a promising intranasal formulation for direct nose-to-brain drug targeting.

## Figures and Tables

**Table 1:** Mixed factorial design for the optimization of RSM-loaded transferosomal formulations.

Independent Variables	Levels
X1:Type of EA	Sodium cholate, sodium deoxycholate, Pluronic^®^ F-68, Pluronic^®^ L-35, Pluronic^®^ L-31, and Span^®^ 60
X2:Cholesterol	Absent Present
Dependent variables	Desirability Constraint
Y1:VS	Minimize
Y2:ZP	Maximize
Y3:%EE	Maximize

Abbreviations: EA, edge activator; VS, vesicle size; ZP, zeta potential; EE, entrapment efficiency.

**Table 2:** Composition and characterization of RSM-loaded transferosomal formulations.

Formulation Code	Factor 1 X1:EA Type	Factor 2 X2:Cholesterol	Response 1 Y1:VS (nm)	Response 2 Y2:ZP (mV)	Response 3 Y3:EE%
F1	Span^®^ 60	Present	491.40 ± 48.79	−32.60 ± 4.94	43.25 ± 5.02
F2	Pluronic^®^ F-68	Present	347.26 ± 56.66	−29.65 ± 0.49	52.33 ± 3.53
F3	Sodium cholate	Present	283.30 ± 8.62	−36.95 ± 1.06	54.80 ± 5.19
F4	Pluronic^®^ L-35	Present	391.95 ± 56.17	−39.60 ± 6.36	51.73 ± 5.46
F5	Pluronic^®^ L-31	Present	387.95 ± 52.82	−30.50 ± 1.27	48.51 ± 1.85
F6	Sodium deoxycholate	Present	248.56 ± 32.47	−35.45 ± 3.18	54.98 ± 6.52
F7	Span^®^ 60	Absent	448.70 ± 14.84	−24.90 ± 1.13	50.68 ± 1.24
F8	Pluronic^®^ F-68	Absent	227.51 ± 13.69	−26.25 ± 4.31	61.37 ± 0.53
F9	Sodium cholate	Absent	208.42 ± 11.95	−33.20 ± 0.28	69.40 ± 0.98
F10	Pluronic^®^ L-35	Absent	276.00 ± 27.05	−29.20 ± 2.26	57.47 ± 2.65
F11	Pluronic^®^ L-31	Absent	352.90 ± 15.41	−30.00 ± 0.42	55.45 ± 5.65
F12	Sodium deoxycholate	Absent	198.63 ± 34.98	−33.45 ± 4.73	95.73 ± 0.09

Abbreviations: EA, edge activator; VS, vesicle size; ZP, zeta potential; EE, entrapment efficiency. All values are represented as mean ± SD (*n* = 3).

**Table 3:** Composition and sol-to-gel transition temperature of the formed in situ gels.

Gel Formulation Code	Composition			
	Pectin (% *w*/*v*)	Pluronic^®^ F-127 (% *w*/*v*)	Pluronic^®^ F-68 (% *w*/*v*)	Sol to Gel Temperature (°C)
G1	0.50	15	0	32.4 ± 0.56
G2	0.50	15	5	36.2 ± 0.28
G3	0.50	15	10	40.1 ± 1.69

**Table 4:** Pharmacokinetic parameters of RSM following the administration of RSM IV aqueous solution and RSM intranasal in situ gel (G2) in plasma and brain tissues.

In Plasma			
Parameter	IV Aqueous Solution	IN In Situ Gel	*p*-Value
C_max_ (ng/mL)	103.30 ± 6.95	14.01 ± 5.30	0.0012
T_max_ (h)	0.25	0.25	-
AUC_0-12h_ (ng.h/mL)	63.40 ± 3.00	27.31 ± 5.60	0.001
Absolute bioavailability (%)	--	43.07%	
In Brain			
Parameter	IV Aqueous Solution	IN In Situ Gel	*p*-Value
C_max_ (ng/mL)	241.93 ± 11.41	381.58 ± 10.15	0.0014
T_max_ (h)	0.25	0.25	-
AUC_0-12h_ (ng.h/mL)	447.08 ± 31.49	586.47 ± 31.50	0.002
Brain bioavailability (%)	--	131.17%	

## Data Availability

The data presented in this study are available in the article.
